# Supplementary figures and images for: Super resolution microscopy and deep learning identify Zika virus reorganization of the endoplasmic reticulum
Source: Sci Rep. 2020 Dec 1;10:20937. doi: 10.1038/s41598-020-77170-3 (PMC7708840; doi:10.1038/s41598-020-77170-3)

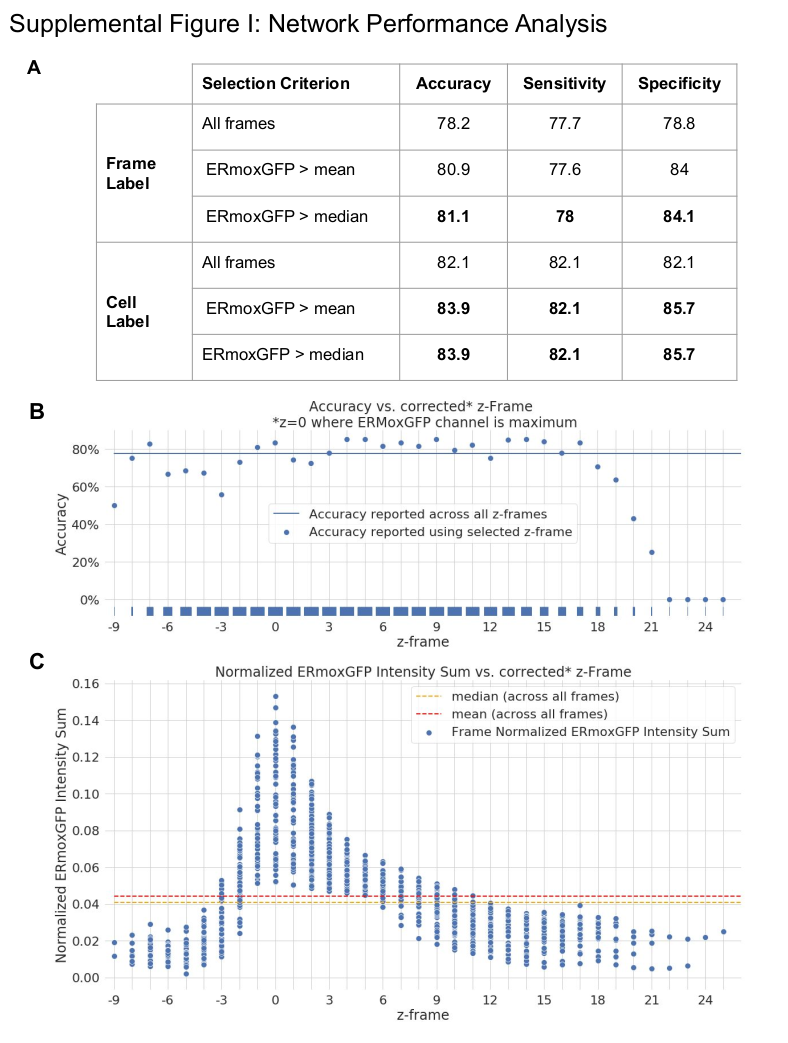

Supplement: Supplementary file 1 — Supplementary Information. [file 41598_2020_77170_MOESM1_ESM.tif]
